# A review of the diagnosis, prevention, and treatment methods of inflammatory bowel disease

**DOI:** 10.25122/jml-2018-0075

**Published:** 2019

**Authors:** Seyed Saeid Seyedian, Forogh Nokhostin, Mehrdad Dargahi Malamir

**Affiliations:** 1. Alimentary Tract Research Center, Ahvaz Jundishapur University of Medical Science, Ahvaz, Iran; 2. Faculty of Medicine, Ahvaz Jundishapur University of Medical Sciences, Ahvaz, Iran; 3. Faculty of Medicine, Medical doctor of Internal Medicine, Ahvaz Jundishapur University of Medical Sciences, Ahvaz, Iran

**Keywords:** Crohn’s disease (CD), Inflammatory Bowel Disease (IBD), ulcerative colitis (UC), IBD treatment

## Abstract

Ulcerative colitis (UC) and Crohn’s disease (CD) are classified as chronic inflammatory bowel diseases (IBD) which have similar symptoms and lead to digestive disorders and inflammation in the digestive system. The reason why they occur is still a mystery. A number of factors can be attributed to the prevalence of CD and UC, some of which include geographical location, inappropriate diet, genetics, and inappropriate immune response. Both diseases are more often diagnosed in urban areas compared to rural areas and both have their own challenges and side effects, but the patients can still have a good quality of life. Given the fact that the prevalence of this disease is higher at younger ages and that it disrupts half the life of the patient, it will, most likely, become a major health problem in the near future, even in developing countries. By reviewing valid scientific resources and evaluating new methods of addressing this disease, the present study aims to provide researchers and patients with new insights into this field and facilitate access to new treatments.

## Introduction

Inflammatory bowel disease (IBD) results from the interaction between genetic and environmental factors which influence the immune responses. Inflammatory bowel diseases are mainly divided into ulcerative colitis (UC) and Crohn’s disease (CD). Crohn’s disease is similar to UC, both of which have been classified as chronic IBD and which cause digestive disorders and inflammation in the gastrointestinal tract. Some of the symptoms of CD and UC include diarrhea, abdominal pain, rectal bleeding, and weight loss. They are mainly characterized by inflammation. Both the diseases may occur in adolescents and adults and affect men and women equally [1]. Despite the similarity between the symptoms of these two diseases, there are some differences between the symptoms of CD and UC.

Crohn’s disease is one of the IBDs that occur in patients between ages 15-35 years. Unlike other inflammatory diseases, IBDs could not be suppressed easily. Consequently, the immune system is stimulated, and part of the intestine is destroyed. It causes pain, diarrhea, fever, and other symptoms. In addition to the serious effect on the lower part of the small intestine, CD can also occur in parts of the digestive tract including the large intestine, stomach, esophagus, or even mouth [2].

Crohn’s disease affects the mouth, anus, and the entire layers of the intestine. Ulcerative colitis affects the mucosal layer of the colon. The lesions occur in the rectum and the intestine. The symptoms are mild to severe and may threaten life [1]. The symptoms of CD and UC are very similar. Malnutrition is very common in CD because the small intestine is responsible for the absorption of nutrients, and CD damages the small intestine [3].

Ulcerative colitis is associated with blood in stool, severe pain, and diarrhea, while in CD there is also a risk of bleeding in severe cases. Rectal bleeding is less common in CD, while UC is commonly associated with rectal bleeding. More than 50% of people with CD suffer from folate and vitamin D deficiency, while more than 50% of people with UC suffer from iron deficiency [4].

The affected areas of the digestive tract vary in these diseases. For example, CD often affects the ileum and a part of the large intestine. It may affect any part of the gastrointestinal tract (GI) including mouth, esophagus, stomach, small intestine, rectum, and anus. In CD, the small intestine often becomes inflamed, while UC is limited to the colon and is found mostly in some parts of the large intestine including colon and rectum. In UC, the large intestine becomes inflamed and the small intestine works naturally [5].

Ulcerative colitis only affects the innermost part of the colon, while CD occurs in all layers of the bowel wall. By understanding the fact thatCD and UC are both major categories of IBD, it could be pointed out that CD can cause serious problems particularly for skin and biliary stones, and UC will be associated with osteoporosis and possibly colon cancer if it lasts over 8–10 years. Crohn’s disease is mostly associated with abdominal pain and problems such as fistula and rectal lesions. In contrast, people with UC usually suffer from intermittent pain consistent with bowel movements [6].

It affects both children and adults. It is estimated that UC affects 2.6 million in Europe and 1.2 million people in North America [7]. Approximately 25% of these patients are diagnosed before the age of 18 years. The disease often begins in adolescence and approximately 25% of patients with IBD are younger than 20 years [8].

Accordingly, clinical, endoscopic, histologic, and radiological tests are used to diagnose UC. About 7-10% of IBDs are unclear [9, 10]. According to literature, UC disease is a mucosal inflammation that is restricted to the colon and frequently expressed precisely by dysentery, abdominal pain, and tenesmus. Inflammatory bowel disease is one of the more frequent forms of dysbiosis diseases. The initial subtype of IBDthat was known as a specialized distinct entity was UC; the term IBD includes the characteristics of both CD and UC. It has long been difficult to distinguish between these two diseases, but now there is a clinical definition for both. Both diseases can affect specific parts of the lives of patients, such as school, job, social life, and family life [11]. Therefore, this review paper aims to investigate the prevalence, causes, diagnosis, and treatment strategies for patients with IBD.

## Reference Reviews

### Introduction and Research Basis

The most common cases of IBD are reported at the age of 15–35 years. According to reports, 25–30% of patients younger than 20 years have been diagnosed with CD, while 20% of patients have been diagnosed with UC [12]. Javier and Pudolovsky reported an increased prevalence of IBD, especially CD, in countries with lower prevalence of the disease. They stated that environmental factors play a key role in the development of this disease. Despite the similarity of the clinical symptoms of the disease in children and adults, problems such as delayed growth have also been reported. Gribovski conducted studies of the possibility of developing an IBD at an early age, pointing out that it is possible to diagnose this disease during adolescence [2]. However, he provided comprehensive reports on cases of CD and UC during infancy.

In a comprehensive study of the CD in Sweden, Askling et al. reported that the disease increased from 2.4-5.4% in every 100 children in 1992–1990 [13]. In contrast, there was no increase in the prevalence of UC. Kugathasan et al. reported that out of every 100.000 children in the state of Wisconsin, 2.56% and 2.14% have been diagnosed with CD and UC, respectively [14]. Similar studies in Iran (1969-2002) showed that the prevalence of IBD has been rising. Mikaeili et al. studied a total of 140 patients in Tehran over a period of 11 years [15]. They reported that most patients with IBD suffered from UC and only a few patients with CD were reported. A comprehensive investigation into the prevalence of IBD in Iran (1973-1982; 1992-2002) reported an increase in the prevalence of UC. It should be pointed out that few people with CD were reported in Iran, emphasizing the lower prevalence of CD [16, 17, 18, 19, 20].

According to Malekzadeh et al., the increased IBD incidende in Iran raised awarness concerning this disease considering changes in lifestyle, diet, increased migration to cities, increased urban population, healthcare improvement and vaccinations. Accordingly, change in diet and health may increase the prevalence of IBD. According to the Cold Chain Theory [21], this change occurred in Iran later than it occurred in developed countries.

### Background

Vahedi et al. (2007) reported that the geographical location can affect the prevalence of IBD. Accordingly, the prevalence of both CD and UC in Africa, Asia, and South Europe was lower than that of Scandinavia, England, and North America. On the other hand, the impact of ethnic and racial characteristics on the prevalence of these diseases cannot be underestimated as the number of reports of this disease has been lower among the blacks [23]. Meanwhile, Mibery and Mann reported that the prevalence of IBD among African-American and non-Black Americans is similar [24]. Acheson conducted comprehensive studies on the effect of geographical location and racial differences on the prevalence of this disease [25]. By examining a wide range of Jewish people, they observed that the prevalence of both CD and UC is higher than that of non-Jewish people. It was reported that although the differences in the prevalence of IBD in different geographic regions emphasize the role of environmental factors, the greater prevalence of this disease among Jews highlights the fact that genetics plays a key role.

According to studies conducted in different regions of the United States (2000–2002),in a total of 1370 newborns and children younger than 17 years, the average time for diagnosis was 10.3 years of age. According to the data, the side effects emerged in 15% of patients before the age of six. Moreover, the side effects were identified in 48% and 37%of patients before 12–6 and 13–17, respectively. The side effects of IBD are more common in children younger than eight years, in comparison with older children. Additionally, the prevalence of UC has been higher than the CD among children younger than eight years [26].

### Causes of the Disease

Although the main cause of the IBD has not yet been fully understood, the comprehensive studies carried out in this regard highlight the role of genetic and environmental factors. Heymen et al. suggested two approaches for the main causes of IBD: 1. Disruption of the mucous system increases the immunological response rate in the human microbiota [27]. 2. Any change in the content of the gut flora or the disruption of the epithelium function stimulates the pathologic response in the normal mucous system. On the other hand, Podolsky pointed out that pathogenicity in inflammatory bowel disease depends on factors such as the patient’s susceptibility, mucosal immunity, and microflora of the intestine [28].

Several researchers have attempted to understand the microorganisms affecting the development of IBD, but no result has been obtained. Meanwhile, microbial flora varies in patients and healthy people. In his microbial culture experiment, Polovsky showed that the levels of Bacteroidetes in patients with CD increased in comparison with healthy people, while lactobacillus and bifidobacterium decreased [28]. According to reports, the levels of enterobacteria increased significantly in patients with CD. On the other hand, patients with CD had higher antibody titers compared to *E. Coli* in healthy people. In a similar study carried out by Dickinson et al., on patients with UC, the side effects of IQA with high aggression and high adhesion were reported in stool specimens [29]. In a comprehensive study, Marteau et al. showed that the levels of *E. Coli* and Bacteroidetes in the normal gut flora were higher in people with IBD [30]. However, some microorganisms such as lactobacillus and bifidobacterium were introduced as useful bacteria which prevent the IBD.

Some reports suggest that the prevalence of IBD does not follow the Mendel’s genetic model. However, various pieces of evidence suggest that the role of genetic factors in the development of these diseases should not be underestimated. For example, various studies have highlighted the role of ethnic-racial differences in IBD, kinship, the prevalence of this disease in twins, chromosomal relationships, hereditary and genetic factors, as well as genetic syndromes. Heymen et al. investigated the effect of genetic factors on the prevalence of IBD [27]. They reported that the risk of these diseases in the first-degree relatives of the patient was 7%, and these individuals are more prone to such diseases. On the other hand, Dickinson et al. reported that in families with a history of IBD, the prevalence of this disease is higher in young people [29]. Similarly, Hayman et al. investigated the effect of genetic relationships between twins on the prevalence of this disease, showing that the effect of genetic relationships among twins was greater in CD than UC [27].

Hample et al. reported that proper nutrition and breast milk can reduce the prevalence of CD and UC because breast milk protects the baby from gastrointestinal infections by helping the development and growth of the gastrointestinal mucosal system [31]. Corra et al. reported that exposure to maternal infections in the fetal or early infancy, as well as newborn babies’ exposure to infections could increase the risk of IBD [32]. They also showed that infectious diarrhea during infancy could increase the prevalence of CD and UC. Koletzko et al. and Montgomery et al. investigated the role of viral infections in the prevalence of IBD [33, 34]. They stated that the measles infection can result in CD. Similarly, Walkkfield et al. carried out a comprehensive study of patients with viral infection in Sweden [35]. They reported that measles increased the prevalence of CD. In contrast, they did not report any increase in the prevalence of UC. However, Ecbum et al by working on perinatal measles infection and subsequent CD, reported that there is no prevalence of measles and CD in England [36]. Several studies were carried out on the role of measles vaccination and its effect on the prevalence of IBD, none of which reported a significant relationship between the two [22].

### Nutrition and Inflammatory Bowel Disease

Given the fact that food first enters the digestive system, it can be said that diet can affect the prevalence of IBD to some extent. Several studies were carried out on the effect of different foods on the prevalence of IBD. For example, Davis et al. investigated the effect of sugar consumption on CD [37]. Some others examined the effect of edible vegetable oils on UC. An investigation into the effects of fatty acid compounds showed that these substances greatly increase the prevalence of IBD [38]. It should be noted that fatty acid compounds consist of acetic acid, tartaric acid, citric acid, and lactic acid. On the other hand, Belluzziet al. studied the effect of fish oil on IBD [39]. They reported that fish oil consumption could be associated with a significant decrease in the clinical signs of IBD because fish oil affects the water-soluble protein molecules (cytokines and leukotrienes).

Smoking can increase the amount of CD4+ T cells which are a type of white blood cell. They can release the inflammatory protein called interferon gamma, which is activated by smoking in the lungs. They move to the intestine and cause inflammation. Smokers are twice as likely to be affected by IBD, compared to other people. Additionally, it could be said that while smoking seems to have harmful effects in CD, but from the epidemiological view it has been overpoweringly proved that smoking protects against UC. However, Corrao et al. [32] reported that smoking possibly has a significantly negative effect on developing UC disease, rather than on CD. On the other hand, studies on the effect of consumption of oral contraceptives have shown that contraceptives increase the prevalence of IBD [40]. The appendix produces antigens that can help increase the immunity of the body against diseases. Appendicitis occurs due to the absorption of gut bacteria in the outer appendix, which increases the infections and appendicitis. Lashner et al. showed that removing the appendix could reduce the risk of UC, particularly after microbial infections [41].

Forbes and Kalantzis investigated the effect of frozen foods in the United States and Europe [42]. They showed that the prevalence of CD in the early twentieth century increased dramatically due to the increased prevalence of refrigerators. Anderson et al. and Forbes et al. showed that the enzymatic activity of psychotropics in refrigerators can contribute to IBD [42, 43]. One of the main causes of food corruption is microorganisms. Enzymes produced in these microorganisms decompose the proteins, fats, and sugars and lead to food corruption. The most dangerous microorganisms are those that are able to survive at the refrigerator temperature. Psychotropics are capable of enzymatic activity at –200–10°C.

### Signs and Diagnosis of the Inflammatory Bowel Disease (IBD)

In a comprehensive study, Hugot et al. investigated the clinical signs and diagnosis of IBD [3]. In order to diagnose IBD, the clinical symptoms of the disease need to be examined. Some of the clinical symptoms of this disease are pediatric growth disorders, anemia, abdominal pain, bloody diarrhea, and arthritis. On the other hand, precise tests are needed to diagnose CD and UC. Common pathogenic bacteria causing IBD are *Salmonella*, *Shigella*, *Yersinia*, *Campylobacter*, *Aeromonas*, *Clostridium Difficile*, *E. coli*, and tuberculosis. Several general assumptions reported by Garcia Rodriguez et al. have recommended that IBD disease develops by way of dysbiosis between protective and harmful bacteria [73]. Patients who suffer from an acute gastroenteritis have been presented with a raised risk of growing IBD. Gradel et al, by working on IBD and disclosing about pathogenic bacteria that can cause gastroenteritis disease such as *Campylobacter* and *Salmonella*, possibly play an essential role in the IBD etiology [74]. In the case of initial rectal bleeding in children, which may occur due to hemorrhoids, polyps, or diverticulum, the possibility of IBD should be reported. CD is far more dangerous than UC; with more than half of the patients with CD suffering from severe infections in both colon and ileum. The most common clinical symptoms of CD include weakness, fatigue, long-term diarrhea with abdominal pain, weight variations, and rectal bleeding. These symptoms should be taken into account. Among people with inflammatory bowel disease, only a few were found to have viral infections in the mouth, stomach, or gastrointestinal tract. Accordingly, the highest number of infections was rectal infections. Burgmann et al. also conducted similar studies to investigate the clinical signs of IBD [4]. The report revealed ulcers in the mouth and gums, chronic infections of the esophagus with pain, and severe swallowing disorders. Few patients had painful stomach ulcers and severe digestive disorders.

In order to accurately diagnose CD and UC, modern medical equipment such as upper gastrointestinal radiography, endoscopy, colonoscopy, and gastrointestinal tract sampling are employed. The above mentioned equipment will help differentiate between these two diseases. However, it is very difficult to diagnose these two diseases in severe cases and in severe infection of the colon. Ulcerative colitis can emerge in various forms, such as inflammation of the rectal lining (proctitis) and panniculitis which occur in 40% and 20% of cases, respectively. In this disease, the results of the colonoscopy test indicate a progressive inflammation that affects the entire region of the rectum and colon. Moreover, some of the obvious clinical symptoms of this condition show that the colonic mucus is red with severe inflammation and small ulcers can be found around the colon. Once UC gets worse, benign tumors (polyps) may develop in the bowel wall. One of the most prominent symptoms of UC severe inflammation of the colonic mucus layers as well as severe inflammation of the rectum, which may extend to the deeper areas of the intestine. It can also affect the colon muscle layer. As a result, the number of intestinal movements decreases. This is known astoxic megacolon which is characterized by a much dilated colon, accompanied by abdominal distension and sometimes fever, abdominal pain, or shock [4, 5].

Some of the main symptoms of CD are mucosal constriction and fistula. Crohn’s disease can cause intestinal mechanical obstructions due to scarring and swelling. Ulcers in the intestinal tract may develop into tracts of their own, known as fistulas. Crohn’s disease can also increase the risk forcolon cancer, which is why people living with the condition must have regular colonoscopies. In order to introduce the best treatment for CD, areas of the body affected by the disease should be identified. For this purpose, radiographic methods can be used to diagnose more precisely the areas of the body affected by CD. Accordingly, CD is different from UC in terms of both medication and surgical treatment. However, in some cases, it is impossible to distinguish between patients with CD and UC. New studies have introduced capsule endoscopy as an important method for diagnosis of CD [6, 44]. Although there is no specific test for the diagnosis of CD or UC, some physical examinations, laboratory examinations, and endoscopy are needed for diagnosis. In some cases, inflammatory bowel disease can be diagnosed by accurate medical examinations such as stool testing, complete blood count (CBC), Barium X-ray imaging, radiological tests, sigmoidoscopy, colonoscopy, upper endoscopy, capsule endoscopy, and some other blood tests. Duigenan and Gee, by working on imaging of pediatric patients with inflammatory bowel disease, stated that CT enterography technique has become the initial imaging technique for appraising IBD and its difficulties in the US recently, because of a combination of rapid scan time, high-resolution evaluation of intestinal and extra-intestinal disease manifestations and 24hours availability in most hospitals [75]. Clinical eye examinations can also play a useful role in diagnosing these diseases. Accordingly, rectal examinations can be performed as well. Thus, in 20-80% of patients with CD, perianal skin tags, itchiness, or pain around the anus may be suggestive of inflammation, fistulization, or abscess around the anal area, which are quite common [45, 46].

In addition to helping diagnose IBD, medical tests help us diagnose which patient needs endoscopy. After observing initial symptoms of the disease, the endoscopic test should be immediately performed to confirm the final diagnosis. It is necessary to examine the fecal calprotecin. Accordingly, an increase in the fecal calprotecin up to 81-91% can be suggestive of developing of the disease. On the other hand, an increase in the fecal calprotecin up to 89-98% can have serious implications [47]. An increase in the lactoferrin up to 80% is alarming, suggesting the likelihood of IBD [48, 49]. In a comprehensive study of the clinical features of UC, Moo et al. reported that some local side effects and external anomalies may appear in the disease [50]. They also reported that severe rectal bleeding, sudden and severe colitis, and colon cancer may emerge. On the other hand, another study showed that the risk of cancer is higher in people with UC who have a history of cancer in their family.

### The Difference between Crohn’s Disease and Ulcerative Colitis

Although there are many differences between CD and UC, both of them are characterized by bowel symptoms which can be seen in 25-40% of patients with IBD [51]. Although most organs are affected by the diseases, symptoms first emerge in the eyes, skin, liver, and joints. The emergence of a symptom outside the bowel increases the risk of development in other organs [20]. Eiden et al. examined other side effects of IBD, pointing out that side effects of CD are more obvious than UC [52]. Some of the side effects of CD are colon bleeding, acute bowel perforation, fistula, abscess, and toxic mega-colon. Nugent and Roy conducted a study on 90 patients with CD [53]. In a comprehensive report, they divided the disease into four stages- mild to moderate, moderate to severe, and chronic severe stage. The patient recovers when the symptoms of the disease disappear as a result of medication or surgical treatments. If medication fails to relieve the clinical symptoms of the disease, the patient will be likely to be at the mild to moderate stage, in which he/she will be able to keep fluids in the intestine but will not suffer from abdominal pain and bowel obstruction. If the disease is not detected or well-treated in the mild to moderate stage, it will progress to another stage (moderate to severe). Those who are at moderate to severe stage may suffer from fever, severe weight loss, acute abdominal pain, intermittent nausea, painful diarrhea (with no symptoms of recovery for up to a week), and anemia. Crohn’s disease has more severe symptoms at the chronic stage, some of which are fever, continuous vomiting, bowel obstruction, abscess, severe abdominal pain, painful diarrhea, and cachexia.

Danovitch investigated 27 patients with ulcerative colitis and described the disease in three stages [54]. The mild stage is associated with rectal bleeding, mild diarrhea less than four-times a day, and mild pain due to proctalgia fugax. If no medication is taken at this stage, the disease will progress to moderate stage. Accordingly, the patient will experience more severe symptoms such as frequent watery diarrhea, painful stool, anemia, mild abdominal pain, and fever. In chronic and severe stages, the patient may suffer from severe abdominal pain, frequent diarrhea up to ten-times a day with severe pain due to proctalgia fugax, increased body temperature (up to 40°C), severe weight loss, and severe anemia.

### History of Disease Treatment

Kamm believed that the main objective of the diagnosis and treatment of the disease is to reduce the symptoms and improve the patient’s health, to completely eliminate the symptoms of the disease or keep the disease at a fixed stage and avoid the surgical treatment [55]. He also stated that in order to treat IBD, it was necessary to determine the type of disease before initiating the treatment. By carefully examining the clinical symptoms of the patient and performing several tests, the severity of the disease, as well as the areas affected by the disease, can be determined. It is important to realize how the body responds to the type of treatment. Inflammatory bowel disease can be treated by a combination of self-care and medical treatments. According to the Food and Drug Administration (FDA), an improved UC is a condition in which all inflammatory symptoms of the bowel, such as bleeding and severe diarrhea, ulcers, proctitis, and colon mucus are improved. Wheeler et al. investigated the diagnosis and symptoms of CD and concluded that unlike improved UC, the only way to detect recovery in CD is to report on improving the patient’s quality of life [56]. They attributed this to the lack of correlation between clinical symptoms of the disease and endoscopic observations.

Feldman et al. conducted research on how to treat CD [57]. The fact that the major causes of CD have remained unknown has hindered the development of strategies and treatments for the disease. Multi-dimensionality of the disease and uncertainty of the severity of the disease have complicated the diagnosis and treatment. Therefore, the objectives of the medical treatment were described as follows:

1. Clinical treatment and improvement of the individual’s clinical condition,
2. Reducing the clinical side effects of the patient,
3. Improving the quality of life,
4. Reducing the drug poisoning,
5. Nutritional support for the patient, and
6. Restricting the patient’s need for admission or surgery.

The first step in treating IBD is pharmaceutical treatments. Physicians usually prescribe medication stage by stage. Firstly, less harmful drugs are prescribed; if these drugs do not provide the desired relief, some other drugs will be prescribed. Accordingly, the corticosteroids, aminosalicylates, antibiotics, supportive medications and immunosuppressive drugs are used to treat IBD. According to the American Therapeutic Association, aminosalicylates can be used to treat improved UC [58, 59]. For mild to moderate disease, aminosalicylates are proper selective drugs that can be used in various forms. The efficacy of aminosalicylates depends on the dosage [60, 62]. Wheeler et al. suggested corticosteroids for moderate to severe chronic conditions in order to improve the symptoms of the disease [56]. Some antibiotics such as amoxicillin, ciprofloxacin, metronidazole, and azithromycin can improve the symptoms of CD. According to Afaf et al., azithromycin and erythromycin ameliorate the extent of colonic damage induced by acetic acid in rats; treatment with azithromycin significantly reduced the seriousness of gross lesions in a dose-dependent manner. On the other hand, erythromycin in small doses had no significant effect while higher doses had a significant effect on intensity of the inflammatory response. The effect of azithromycin is nearly doubled when compared to the corresponding doses of erythromycin used in their study. Also, treated rats showed a faster weight recovery as compared to the acetic acid control group. However, they cited that patients with resistance to antibiotic treatment can take corticosteroid drugs and immune regulators. Hanauer and Stromberg reported that drugs used to treat oral lesions in CD include sucralfate, carboxymethylene glucose, or hydrocortisone [62]. On the other hand, Masuri et al. emphasized the effect of using drug compounds containing living microorganisms (probiotics) on the reduction of bowel wall inflammation [63]. Methotrexate can also be used in patients who cannot tolerate azathioprine and mercaptopurine. However, surgical treatment can be used in cases where drug treatments do not improve the symptoms of IBD. Masuri et al. introduced scientific methods of treatmentfor CD and UC in a schematic diagram and classified table [63].

Jorgensen et al. reported that drug treatment in CD depends on the location of inflammation, the severity of the disease, the side effects of the disease, and the patient’s response to drug treatment [65]. Over the past decades, various drug treatments have been developed for this disease. The type of these drugsvaries, andthey perform differently in enhancing the patient’s immune system. However, the drug treatments increase the likelihood of adverse effects in the patient. Therefore, the main approach for the treatment of CD is to prescribe weaker drugs. Then, stronger drugs can be prescribed for the patient. Sulfasalazine, which contains 5-ASA, was the first aminosalicylate used to treat CD. This drug could improve the clinical symptoms of patients with mild to moderate Crohn’s disease. Unfortunately, this drug was associated with side effects. However, patients who suffered from inflammation of the small intestine or underwent surgery were less likely to benefit from these drugs [65].

Mesalamine as a nonsteroidal anti-inflammatory drug with drug class of 5-aminosalicyclic-acid derivative has been used for a long time and has a powerful effect in the remedy of IBD. As showed recently, this drug is really useful, well-tolerated and a safe drug for the remedy of ordinary ulcerative colitis that applied in most patients who suffer this disorder. One of the oldest remedies presently used in the management of IBD are aminosalicylates. Salazopyrin is the original drug in this category, but mesalazine (5-aminosalicylic acid [5-ASA]) is the active mediety drug of this category and is the principal aminosalicylate used in IBD remedy nowadays [77].

**Figure 1:**
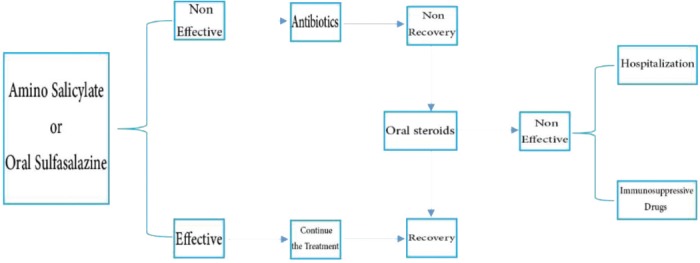
Treatment proposed by Mesuri et al. (1994) for Crohn’s disease.

**Table 1:** Treatment proposed by Mesuri et al. (1994) for ulcerative colitis

**Mild to moderate**	
**Distal colitis**	Oral or rectal aminosalicylates (suppository), rectal corticosteroid
**Extensive colitis**	Oral aminosalicylates
**Medium to severe**	
**Distal colitis**	Oral corticosteroid, rectal corticosteroid
**Extensive colitis**	Oral corticosteroid
**Chronic or critical**	
**Extensive colitis**	Intensive corticosteroid colitis, intravenous cyclosporins
**Recovery**	
**Distal colitis**	Oral or rectal aminosalicylates, oral azathioprine or mercaptopurine
**Extensive colitis**	Oral aminosalicylates, oral azathioprine or mercaptopurine

The initial stage of IBD remedy is by applying anti-inflammatory drugs. Frequent remedies in this group include; Corticosteroids and Oral 5-aminosalicylates. Oral 5-aminosalicylates include sulfasalazine (Azulfidine), which contains sulfa, and mesalamine (Asacol HD, Delzicol, others). Oral 5-aminosalicylates have been widely used in the past but now are generally considered of limited benefit. On the other hand, corticosteroids such as prednisone and budesonide (Entocort EC) can help reduce inflammation in your body, but they don’t work for everyone with CD. Doctors generally use them only if you don’t respond to other treatments. These drugs work through direct contact with the inflammatory tissue. The possible side effects of anti-inflammatory drugs include nausea, vomiting, heartburn, diarrhea, and headache. Omega-3 fatty acids are also anti-inflammatory agents that can contribute to the treatment of IBD. Pentasa and Asacol are also used in the treatment of patients with CD and ileus colitis. Accordingly, Rowasa plays an important role in the treatment of patients with prostate cancer. Physicians usually start with Pentasa or Asacol for ileum colitis. If Pentasa or Asacol is fruitful, physicians may prescribe antibiotics such as Cipro or Flagyl for long periods (often several months). However, evidence suggests that antibiotics do not perform well in the treatment of CD [66]. Siegel suggested that each patient should be treated separately because the type of disease and the clinical symptoms may vary from patient to patient [67]. On the other hand, each patient with CD is familiar with symptoms, risk taking in treatment, fear of disease, morale, and treatment based on his/her personal experience. The general drugs for the treatment of CD are aminosalicylate, antibiotics, boneside, systemic corticosteroids, tyopurines, methotrexate, and TFT. These drugs can be used alone or in combination to optimize the treatment.

Tanida et al. studied drug treatments for UC [68]. They reported that the conventional treatments for UC include amniosalysilic-5, corticosteroids, and purine analogues (azathioprine and morapopurine- 6). They introduced anti-inflammatory cytokines such as TNF-α (tumor necrosis factor-α), interleukin 2, and integrin α4β7 as the main treatment for this disease. However, patients who cannot respond to conventional treatments are treated with other drugs such as calicineurin inhibitors, tacrolimus, TNF-α inhibitors, and a neutral antibody (vedolizumab). Tanida et al. pointed out that the treatments proposed for ulcerative colitis may be useful, but they are generally not effective [68]. These treatments, while improving the early symptoms of the disease, may worsen the symptoms in the long run. Therefore, Tanida et al. suggested A2(ANX) as a new molecular treatment strategy for the prevention of TNF-α destruction that could control ulcerative colitis [68].

Metronidazole is one of the antibiotic drugs used in IBD, usually after surgical treatment or in cases where the side effects of inflammation appear in the body. Cyclosporine is the most commonly used drug in the treatment of ulcerative colitis. It should be pointed out that this drug can be toxic for patients with CD due to the need for higher doses. One of the most effective medications used in the treatment of UC is corticosteroid. However, body resistance to these drugs makes it difficult to take these drugs. Van Assche, Vermeire, and Rutgeerts reported that if UC does not respond to corticosteroid injections, it will be necessary to use cyclosporine, complete colectomy, or infliximab based on the patient’s clinical condition and radiographic and laboratory findings [69]. Similarly, Cima and Pemberton reported that sometimes patients with coronary fistula, CD, and UC do not respond to conventional treatments. In such cases, infliximab is used to treat the diseases [70].

It is said that special treatment course can improve the symptoms of IBD in the short term. However, this method cannot improve and reduce the symptoms of the disease in long-term treatment. Corticosteroids can contribute to the treatment of CD. Accordingly, Zachos et al. reported that low-fat diets did not improve or reduce the symptoms of CDCD [71]. Middleton et al. reported that minerals, proteins, liquids, vitamins, and sugar-free substances could relieve the side effects of IBD [72]. On the other hand, it is stated that fruits, liquids, and fibers can reduce the risk of constipation in patients with UC.

## Results and Conclusion

The number of people with IBD is increasing rapidly. Researchers are trying to find new treatments to eliminate the disease and improve the resulting complications. IBD is a debilitating disorder that causes serious implications for the patients. It affects the general health and quality of life of the patients. Inflammatory bowel disease has recently become a serious challenge in medical science. Given the fact that several studies shave been carried out on adult patients, it seems researchers can investigate this disease in children as well, in order to offer the best way to treat it. In spite of comprehensive research on the recognition, diagnosis, and treatment of IBD, there are still some ambiguities. Given the prevalence of this disease in developing countries, including Iran, further studies are needed to examine the causes, diagnosis, and treatment of these diseases. Researchers aim to obtain more accurate data on the mortality rate and the prevalence of this disease. It can be concluded that all diagnostic methods should first be considered in order to treat this disease. The results showed that foods do not cause the disease, but some foods worsen the symptoms. Some of the most common foods that worsen the symptoms include alcohol, coffee, soft drinks, spicy foods, beans, fatty foods, nuts, seeds, unrefined fruits and vegetables, red meat, and dairy products. On the other hand, learning stress management techniques can help improve IBD.

## Conflict of Interest

The authors confirm that there are no conflicts of interest.
